# Community Analysis and Recovery of Phenol-degrading Bacteria from Drinking Water Biofilters

**DOI:** 10.3389/fmicb.2016.00495

**Published:** 2016-04-12

**Authors:** Qihui Gu, Qingping Wu, Jumei Zhang, Weipeng Guo, Huiqing Wu, Ming Sun

**Affiliations:** ^1^School of Bioscience and Bioengineering, South China University of TechnologyGuangzhou, China; ^2^Guangdong Institute of Microbiology, State Key Laboratory of Applied Microbiology Southern China, Guangdong Provincial Key Laboratory of Microbial Culture Collection and Application, Guangdong Open Laboratory of Applied MicrobiologyGuangzhou, China

**Keywords:** phenol, biodegradation, denaturing gradient gel electrophoresis, metabolic pathway, drinking water

## Abstract

Phenol is a ubiquitous organic contaminant in drinking water. Biodegradation plays an important role in the elimination of phenol pollution in the environment, but the information about phenol removal by drinking water biofilters is still lacking. Herein, we study an acclimated bacterial community that can degrade over 80% of 300 mg/L phenol within 3 days. PCR detection of genotypes involved in bacterial phenol degradation revealed that the degradation pathways contained the initial oxidative attack by phenol hydroxylase, and subsequent ring fission by catechol 1,2-dioxygenase. Based on the PCR denatured gradient gel electrophoresis (PCR-DGGE) profiles of bacteria from biological activated carbon (BAC), the predominant bacteria in drinking water biofilters including *Delftia* sp., *Achromobacter* sp., and *Agrobacterium* sp., which together comprised up to 50% of the total microorganisms. In addition, a shift in bacterial community structure was observed during phenol biodegradation. Furthermore, the most effective phenol-degrading strain DW-1 that correspond to the main band in denaturing gradient gel electrophoresis (DGGE) profile was isolated and identified as *Acinetobacter* sp., according to phylogenetic analyses of the 16S ribosomal ribonucleic acid (rRNA) gene sequences. The strain DW-1 also produced the most important enzyme, phenol hydroxylase, and it also exhibited a good ability to degrade phenol when immobilized on granular active carbon (GAC). This study indicates that the enrichment culture has great potential application for treatment of phenol-polluted drinking water sources, and the indigenous phenol-degrading microorganism could recover from drinking water biofilters as an efficient resource for phenol removal. Therefore, the aim of this study is to draw attention to recover native phenol-degrading bacteria from drinking water biofilters, and use these native microorganisms as phenolic water remediation in drinking water sources.

## Introduction

Phenol is extensively used in various organic compounds, such as agricultural chemicals, pesticides, dyes, and drugs (Basha et al., 2010; Zhang et al., 2013). However, because of its carcinogenic and toxic properties, phenol poses risks to human health and other forms of life (Kumar et al., 2005; El-Naas et al., 2009). Phenol contaminants at high concentration have been detected in the environment (50–1500 mg/L; Cooper and Nicell, 1996; Adak et al., 2006) and in drinking water (0.21 to1130 mg/L; Baker et al., 1978; Jarvis et al., 1985). The United States Environmental Protection Agency (US EPA) deemed phenol a priority pollutant in 1979. In the 2006 standards for drinking water quality produced by the Ministry of Public Health of China, the maximum volatile phenol level permitted in drinking water is 2 ppb. The elimination of phenol from drinking water is thus a critical issue in China today.

For several years, biological processes have proven to be effective and eco-friendly tools for remediating contaminated sites compared with traditional physical and chemical methods. Numerous phenol-degrading microorganisms have been discovered, including *Pseudomonas putida* (Abu Hamed et al., 2004), *Gliomastix indicus* (Singh et al., 2008), *Sphingomonas chlorophenolica* (Nair et al., 2008), *Bacillus brevis* (Arutchelvan et al., 2006), and *Cyanobacterium synechococcus* (Song et al., 2009). However, most of these microorganisms are isolated from wastewater, soil, activated sludge, or other nutrient-rich conditions, which are not suitable to apply under micropollution environment. As we all know, drinking water biofilters are used to treat organic micropollution in drinking water treatment system, and they usually perform well in reducing natural organic matter (NOM), disinfection by-products and odor and taste compounds (Eighmy et al., 1992; Feng et al., 2013; Liao et al., 2015a). However, to our knowledge, direct information on phenol removal from drinking water biofilters by indigenous microorganisms is still lacking. In addition, the knowledge of the change in microbial communities in drinking water biofilters exposed to phenol is also limited, which inhibits the isolation and identification of indigenous microorganisms which can effectively degrade phenol. Moreover, the rates and mechanisms of phenol degradation by degraders at low concentrations are unknown. Therefore, it is necessary to study the potential of drinking water biofilters for phenol biodegradation and recover phenol-degrading bacteria from biofilters for bioremediation.

In this study, the biological activated carbon (BAC) was sampled from a drinking-water plant in Guangzhou City of South China and found to be enriched with a phenol-degrading bacterial community. The differences in the microbial community structure before and after phenol acclimation were examined using the molecular biology tool PCR-DGGE. In order to determine the biodegradation mechanisms of the phenol-degrading enrichment culture, the major functional genes in the phenol-degradation pathways were also investigated. Finally, the strain that could most effectively eliminate low concentration of phenol was isolated and characterized. Meanwhile, the recovery of phenol-degrading microorganism immobilized on granular active carbon (GAC) for being used as phenolic water remediation in drinking water sources was investigated. The findings of the present study provide new insight into the evolution of bacterial communities that during phenol degradation processes, and also provide information on the phenol-degrading bacteria which was isolated from drinking water biofilters. The aim of this study is to draw attention to recover native phenol-degrading bacteria from drinking water biofilters, and apply these native microorganisms to remove phenol from drinking water sources.

## Materials and Methods

### Acclimating Phenol-degrading Bacterial Community

Four BAC samples were obtained from two BAC filters at different depths of the activated carbon filter layer (0 and 50 cm) in January 2014. The two BAC filters were from a stably operating drinking water treatment plant (DWTP; both with surface river water as source water) in Guangzhou city, South China. The source water contained approximately 5.8 mg/L dissolved oxygen (DO), 0.12 mg/L ammonia (NH_3_-N), 2.4 mg/L chemical oxygen demand (COD), 50 NTU turbidity and 4 μg/L volatile phenols. The drinking water treatment process of the DWTP including prechlorination, coagulation, and sedimentation, ozonation, BAC filtering and disinfection (with chlorine). The site was chosen, it is because the source water has been contaminated by phenol. Thus, the removal of phenol is urgently needed. In addition, the DWTP supply approximately one million m^3^/d of water for approximately 350,000 local residents (Chang-jun, 2005). Besides, the DWPT own the advanced ozone-activated carbon adsorption technology. Therefore, abundant microbes existed in the BAC layer, which allow for screening functional microorganisms. In order to isolate bacteria with the potential that could endure and degrade high concentrations of phenol, phenol enrichment was conducted. Firstly, microcosm samples A, B, C, D were prepared by using four BAC samples. Samples A and B were collected from one BAC filter at the depth of 0 and 50 cm, respectively. Similarly, Samples C and D were from the other BAC filter. Briefly, 10 g of BAC sample was put into a 250 mL Erlenmeyer flask containing 100 mL of mineral salt medium (MSM) that supplemented with 50 mg/L of phenol as the sole carbon source. The composition of MSM including (mg L^-1^): MgSO_4_·7H_2_O, 0.1; NaCl, 0.2; NH_4_Cl, 0.5; Na_2_HPO_4_·12H_2_O, 0.5; KH_2_PO_4_, 0.5; FeCl_3_·6H_2_O, 0.1; and CaSO_4_·H_2_O, 0.1. Subsequently, the enrichment flask was incubated at 30°C and shaken at 120 rpm in an environmental chamber in the dark. After 8 days, 1 ml of the acclimation sample was withdrawn at regular time intervals, and added it into a new MSM supplemented with a higher concentration of phenol. This procedure was repeated nine times over a period of 50 days. During the enrichment, the concentration of phenol in the MSM was investigated. The removal efficiency formula was (C_0_–C_n_)/C_0_, where C_0_ is the initial concentration of phenol, and C_n_ is the final concentration of phenol.

### Total DNA Extraction, DGGE Analysis and Sequencing

The total genomic DNA of four initial BAC samples that before phenol acclimation was extracted using a soil DNA extraction kit (Sangon Biotech, Shanghai, China). The extracted products were visualized on a 2% agarose gel. By the end of phenol acclimation, the DNA of the four enrichment cultures was also extracted. All of the extracted DNA were amplified using the variable V3 region of the 16S ribosomal ribonucleic acid (rRNA) gene universal primers 341f, 5′-CCTACGGGAGGCAGCAG-3′; 341f-GC, GC-clamp with 5′-CGCCCGCCGCGCGCGGCGGGCGGGGCGGGGGCACGGGGGG-3′ attached to the 5′ end of 341f; and 518r, 5′-ATTACCGCGGCTGCTGG-3′ (Muyzer et al., 1993; Hesham et al., 2011). Takara Green Master Mix (Takara, Dalian, China) was used for PCR, and the amplification was performed on the PCR amplifier (Bio-Rad, Hercules, CA, USA). The final volume of 24 μL that included Green Master Mix (12.5 μL), primers (0.5 μL each), the DNA template (2 μL) and ddH_2_O (8.5 μL). In order to further optimize the amplification settings and limit the formation of spurious by-products, a PCR touchdown test was performed, and this procedure was described as previously (Jiang et al., 2010). The PCR products were analyzed using the DCode^TM^ detection system by DGGE (Bio-Rad Laboratory, Hercules, CA, USA). Samples that contained nearly equal quantities of amplicons were added to the polyacrylamide gel with 40 to 70% denaturing gradient that composed of urea and formamide (100% corresponding to 7 M of urea and 40% v/v formamide). Electrophoresis was performed at 30 V and 60°C for 30 min, and then at 80 V for 750 min. Following electrophoresis, the gels were incubated in Milli-Q for 5 min and then rinsed twice. The gels were stained with goldview for 30 min on a reciprocating table, and then be visualized using a UV transilluminator gel imaging system (GE Healthcare, Milwaukee, WI, USA). The obtained DGGE profiles were analyzed by clustering via the unweighted pair group method with arithmetic mean (UPGMA) and principal component analyses (PCA) using the Quantity One software package (Bio-Rad). The dominant bands were excised with a sterile razor blade and incubated in 100 μL of sterile water at 4°C overnight, which dissolved the DNA in the bands. Two microliters of the overnight solution were used as the template for the PCR with the 341f and 518r primers under the conditions that described as above. The PCR products were purified using an agarose gel DNA purification kit (Magen, Guangzhou, China). The products were then sequenced by Sangon Biotech. In order to determine phylogenetic affiliations, the resulting sequences were compared with the National Center for Biotechnology Information (NCBI) GenBank database using the basic local alignment search tool (BLAST). A phylogenetic analysis of the sequence data was performed on the MEGA 6.06 software package.

### Isolation of Phenol-degrading Bacteria and Phenol Degradation Tests

Enrichment culture was used to isolate phenol-degrading bacteria. In order to isolate phenol degraders from the final enrichment culture, the enrichment culture was diluted and spread onto MSM agar supplemented with 300 mg/L of phenol as the sole carbon and energy source with a sterile rod. The plate was incubated at 30°C for 48 h. After the growth period, a single colony with distinct morphological characteristics was isolated and streaked on the same medium, and a single colony was again selected until a pure culture was obtained. Subsequently, the phenol degradation capacities of the isolates were evaluated. Isolates were firstly incubated in an R2A (Massa et al., 1998) fluid nutrient medium for 24 h, and then the cell suspensions were pelleted by centrifugation at 5,000 × *g* for 10 min. The supernatant was then discarded. MSM without phenol was used to wash the cells three times. The cells were then re-suspended in a 200-mL Erlenmeyer flask that contained 80 mL of MSM supplemented with 500 μg/L of phenol as a carbon and energy source. Samples of the MSM solution were collected at appropriate intervals for phenol quantification. Prior to phenol quantification, the cell suspensions were separated by centrifugation at 12,000 rpm for 10 min. The supernatant was filtered through a 0.22-μm-pore-size filter for subsequent phenol quantification. The entire operation was completed under sterile conditions. Finally, the most efficient phenol-removing strain was determined based on the amount of remaining phenol. Subsequently, the phenol-degrading capacity of the most efficient phenol-removing strain immobilized on GAC was also evaluated. The BAC samples and sand filtered water used for the experiment were obtained from stable operation DWTP as described in section “Acclimating Phenol-Degrading Bacterial Community.” Six glass columns (inner diameter = 40 cm) with a working volume of 450 mL were filled with the obtained BAC samples which were sterilized before. Three of the BAC columns were used as control, the other three samples were immobilized with phenol degrader which was isolated from enrichment culture. Sand filtered water samples with different initial phenol concentrations of 50, 250, and 500 μg/L were operated using peristaltic pumps (Lange, Beijing, China), and the empty bed contact time (EBCT) was 18 min. The whole water treatment process was maintained at room temperature. The eﬄuent water with different initial phenol concentrations were collected for phenol detection after operation for 1 h. The determination of phenol concentration was performed by HPLC (Agilent 1200, USA) using a C18 column (250 mm × 4.6 mm i.d., 5 μm, Agilent, USA) maintained at 30°C. The HPLC separation was carried out using methanol-water (50:50, v/v) as the mobile phase at a flow rate of 0.5 mL/min. Phenol was detected by a diode array detector (DAD) at a wavelength of 270 nm. The retention time of phenol was 11.6 min under the above conditions. Quantification was conducted by integration of the peak areas following the external standards.

For bacterial identification, the phenol degrader was incubated in a R2A fluid nutrient medium for 12 h, and the DNA was extracted following the bacterial DNA extraction kit instructions (Dongshen Biotech, Guangzhou, China). The 16S rRNA gene was amplified using bacterial universal primers 27f (5′-GTGCTGCAGAGAGTTTGATCCTGGCTCAG-3′) and 1492r (5′-CACGGATCCTACGGGTACCTTGTTACGACTT-3′) (Dymock et al., 1996). The PCR products were then sequenced (Sangon, Shanghai, China). After editing and verifying the sequences manually, the closest relatives of the most common one were identified using the BLAST software in the GenBank database of NCBI^1^ Phylogenetic trees were constructed as described previously (Jin et al., 2013).

### PCR Detection of Key Functional Genes in Phenol-degradation Pathways

Genomic DNA was extracted from the phenol-enriched culture that had the highest degradation rate and formed reference strain DW-1. To study the phenol-degradation pathways, detection of the key functional genes in the biochemical pathway was performed via PCR using primers for phenol hydroxylase(Lph), catechol 1,2-dioxygenase (1,2-CTD), catechol 2,3-dioxygenase (2,3-CTD), and aromatic hydroxylase α-subunits (TBMD). The genes encoding these enzymes were amplified by using the primers sets: Lphf (5′-CGCCAGAACATTTATCGATC-3′), Lphr (5′-AGGCATCAAGATCACCGACTG-3′) (Xu et al., 2001); 1,2-CTDf (5′-ACCATCGARGGYCCSCTSTAY-3′), 1,2-CTDr (5′-GTTRATCTGGGTGGTSAG-3′); 2,3-CTDf (5′-GARCTSTAYGCSGAYAAGGAR-3′), 2,3-CTDr (5′-RCCGCTSGGRTCGAAGAARTA-3′) (Sei and Fathepure, 2009); and TBMDf (5′-CGCCAGAACCACTTGTCRRTCCA-3′), TBMDr (5′-ACCGGGATATTTYTC TTCSAGCA-3′) (Hendrickx et al., 2006). PCR amplifications were performed using a 24-μL mixture of 12.5 μL PCR Taq-mix, 0.5 μL of each primer, 2 μL of template and 8.5 μL of dd H_2_O. The amplification conditions were as follows: initial denaturation at 94°C for three min; 35 cycles of 94°C for 50 s, 56°C for 30 s, and 72°C for 3 min; and a final extension phase at 72°C for 10 min.

### Nucleotide Sequence Accession Number

The nucleotide sequences determined in this study have been deposited in the GenBank database. The accession numbers for the nucleotide sequences obtained from the DGGE bands are KU375555-KU375568, and for functional gene are KU512639, KU512641, KU512638, KU512640. The accession number for the 16S rRNA gene sequences of strain DW-1 is KU499947.

## Results

### Acclimating Phenol-degrading Bacterial Community

**FIGURE 1:**
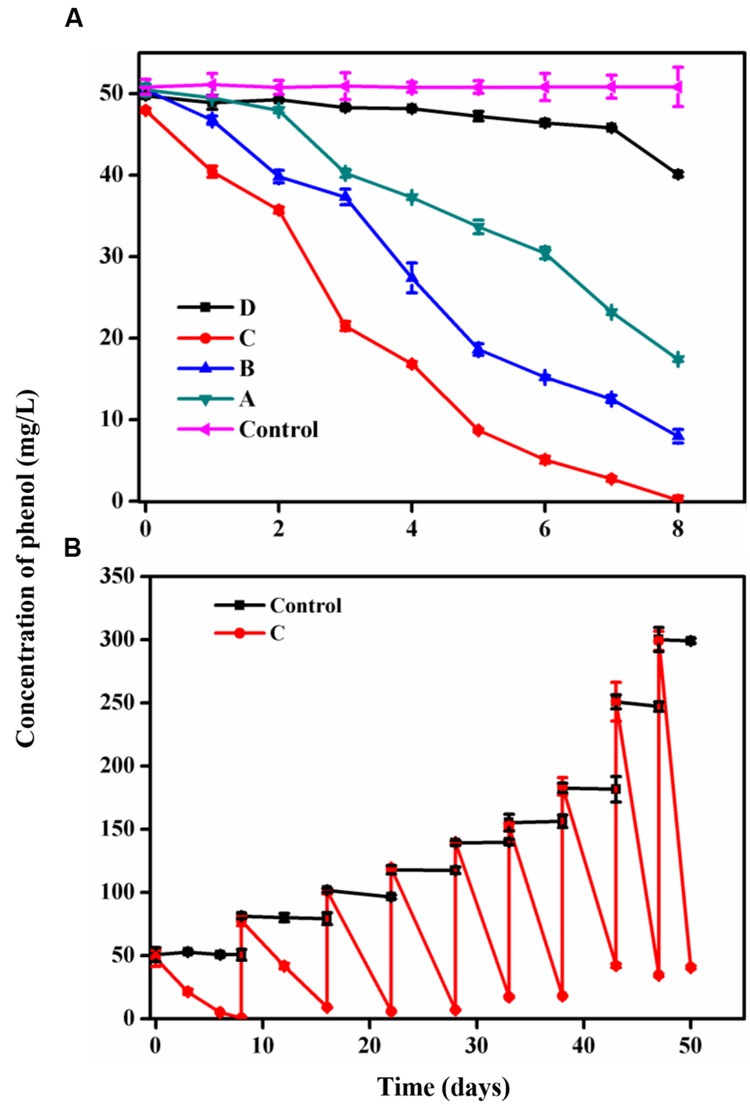
**(A)** Phenol biodegradation in the four microcosms during 8-day acclimation. **(B)** the sample C were continuously acclimated with different concentrations of phenol in 50 days. No phenol degradation occurred in autoclaved control bottles. The mean values for three active bottles and three control bottles were collected.

Four samples were acclimated for 8 days. **Figure 1A** shows the samples A, B, and C, which exhibited rapid phenol-degrading ability, especially sample C that could degrade 92% of phenol at a concentration of 50 mg/L in 8 days. Therefore, sample C was chosen for focused investigation. **Figure 1B** shows the enriched sample C that was exposed to phenol concentrations of 50 to 300 mg/L for almost 2 months. The other three samples were acclimated for 50 days along with sample C (Supplementary Figure S4). At the begin of acclimation period, 50 mg/L of phenol was almost completely degraded over 8 days, and 80 mg/L of phenol was almost completely degraded after 8 days by continuous acclimation. The final acclimated community could degrade over 80% in 300 mg/L of phenol within 3 days. The biodegradation of phenol by a microbial community that obtained from drinking water BAC filters has not been reported previously. In addition, the time of the degradation process was shortened by acclimation, even though the concentration of phenol was increased from 50 to 300 mg/L. Thus, the enrichment culture in this study has ability to remove phenol from drinking water source. Moreover, the phenol-degrading bacteria are more likely to be isolated from the enrichment culture.

### Characterization of the Bacterial Community Structure

**FIGURE 2:**
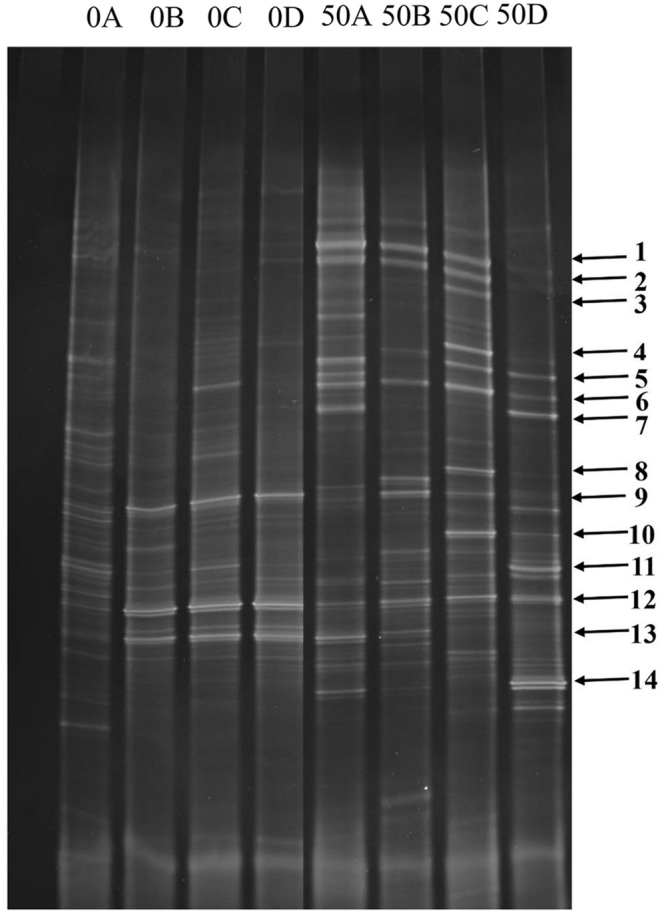
**Denaturing gradient gel electrophoresis (DGGE) profile of amplicons of V3 partitial sequence of bacterial 16S rDNA from BAC samples before phenol acclimation (0A, 0B, 0C, and 0D) and from the final enrichment cultures (50A, 50B, 50C, and 50D)**.

**FIGURE 3:**
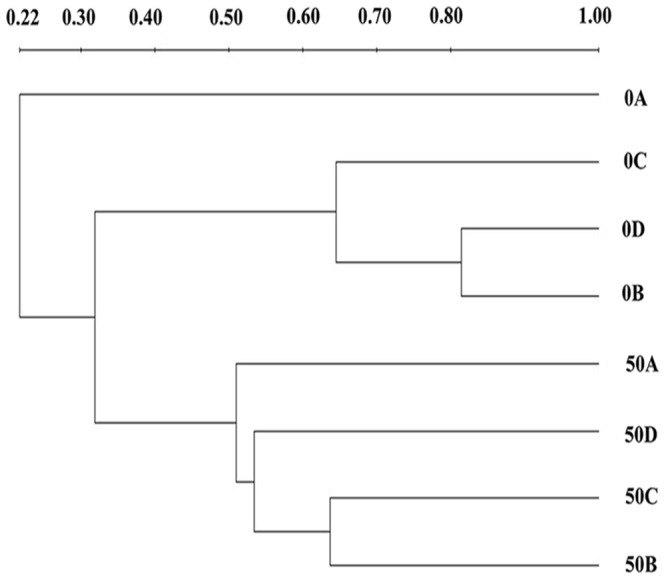
**Unweighted pair group method with arithmetic mean (UPGMA) cluster analysis of the relatedness of PCR denatured gradient gel electrophoresis (PCR-DGGE) banding patterns from BAC samples before before phenol acclimation (0A, 0B, 0C, and 0D) and from the final enrichment cultures (50A, 50B, 50C, and 50D)**.

**FIGURE 4:**
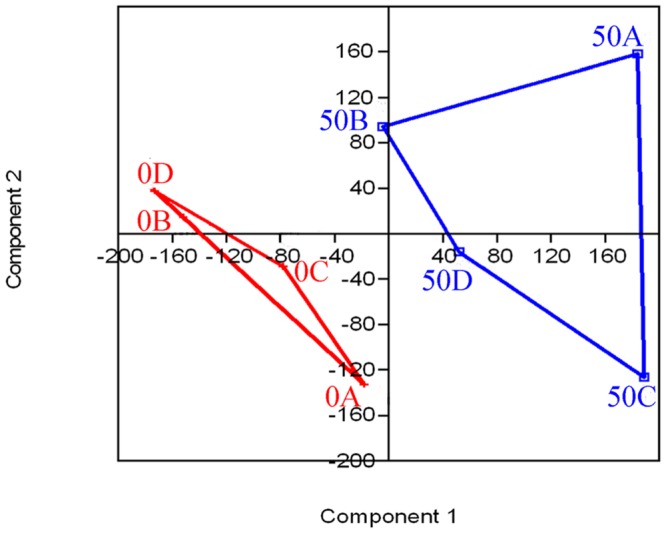
**Principal component analysis of the relatedness of PCR-DGGE banding patterns from BAC samples before phenol acclimation (0A, 0B, 0C, and 0D) and from the final enrichment cultures (50A, 50B, 50C, and 50D)**.

As shown in **Figure 2**, before acclimation, species corresponding to bands 5, 6, 7, 9, 12, and 13 (especially bands 9, 12, and 13, which appeared at almost all depths of the BAC layer, and thus correspond to the dominant microbial community) prevailed throughout the acclimation period. These species were 98, 85, 97, 99, 99, and 99% similar to *Campylobacter* sp., *Niastella* sp., *Deinococcus* sp., *Delftia* sp., *Achromobacter* sp. and *Agrobacterium* sp., respectively. After the acclimation period, additional bands (1, 2, 3, 4, 8, 10, 11, and 14) were formed, and represented the dominant bacteria. Through sequencing, these bands were correspond to *Sulfurospirillum* sp., *Rhodospirillum* sp., *Acidovorax* sp., *Acinetobacter* sp., *Sphingobium* sp., *Thiomonas* sp*., Pseudomonas* sp., and *Comamonas* sp., respectively. These results are presented in **Table 1**. The UPGMA cluster analysis results are presented in the **Figure 3**, we can see that all of the samples generated a coherent cluster with similarity indices exceeding 60% before phenol acclimation, except for 0A. Additionally, the profiles of samples 50A, 50B, 50C, and 50D clustered closely together, with similarities as high as 50%. Interestingly, the samples before phenol acclimation and after 50 days acclimation showed the low similarities of 22%. Similarly, it can also be seen from **Figure 4** that the samples before phenol acclimation and after 50 days acclimation were clustered in two different areas, which revealed great variations between the samples before and after acclimation.

**Table 1:** Phylogenetic sequence affiliation and association with the closest related amplified 16S r DNA gene sequences excised from denaturing gradient gel electrophoresis (DGGE) gels.

Bands^a^	Identity (%)	Closest relative	Phylogenetic affiliation	Accession number
**1**	87	*Sulfurospirillum arcachonense* DSM (JFBL01000027)	*Epsilonproteobacteria*	KU375555
**2**	98	*Rhodospirillum rubrum* F11 (CP003046)	*Alphaproteobacteria*	KU375556
**3**	94	*Acidovorax avenae* sub sp.(CP002521)	*Betaproteobacteria*	KU375557
**4**	99	*Acinetobacter oleivorans* (CP002080)	*Gammaproteobacteria*	KU375558
**5**	98	*Campylobacter hominis* ATCC BAA-381(CP000776)	*Epsilonproteobacteria*	KU375559
**6**	85	*Niastella koreensis* GR20-10 (CP003178)	*Bacteroidetes*	KU375560
**7**	97	*Deinococcus geothermalis* DSM 11300 *plasmid* pDGEO01(CP000358)	*Deinococcus-Thermus*	KU375561
**8**	93	*Novosphingobium* sp. PP1Y (FR856862)	*Alphaproteobacteria*	KU375562
**9**	99	*Delftia* sp. Cs1-4 (CP002735)	*Betaproteobacteria*	KU375563
**10**	98	*Thiomonas* sp. 3As (FP475956)	*Betaproteobacteria*	KU375564
**11**	100	*Pseudomonas monteilii* SB3101 (CP006979)	*Gammaproteobacteria*	KU375565
**12**	99	*Achromobacter xylosoxidans* A8 (CP002287)	*Betaproteobacteria*	KU375566
**13**	99	*Agrobacterium fabrum* str.C58 (AE007870)	*Alphaproteobacteria*	KU375567
**14**	100	*Comamonas testosteroni* CNB-2 (CP001220)	*Betaproteobacteria*	KU375568

*^a^The band labels correspond to those from the DGGE gels shown in **Figure 2**.*

### Isolation and Identification of Phenol-degrading Bacteria

**FIGURE 5:**
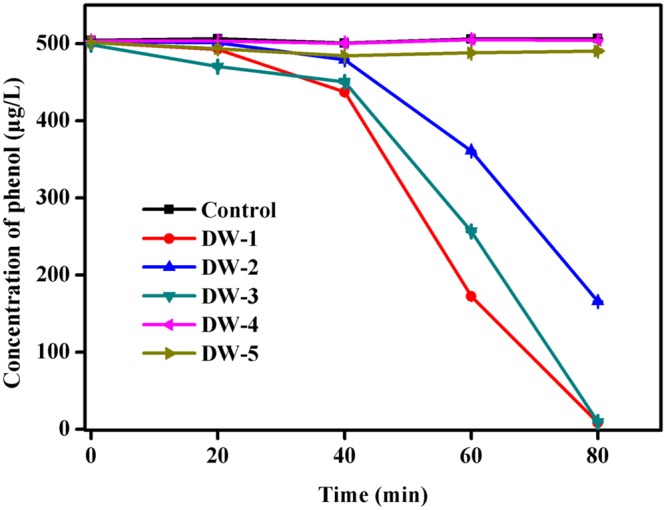
**Phenol biodegradation by the five isolates designated as DW-1, DW-2, DW-3, DW-4, and DW-5.** Cultures were incubated in 80 mL of MSM supplemented with 500 μg/L of phenol at 30°C at 120 rpm. No phenol degradation occurred in the autoclaved control bottles. The mean values from triplicate experiments and the standard errors of the means, indicated by error bars, are shown.

Following acclimation, five isolates were obtained from the final enrichment sample C. These isolates were coded as strains DW-1, DW-2, DW-3, DW-4, and DW-5, and their phenol removal capabilities are shown in **Figure 5**. The strains DW-1, DW-2, and DW-3 were able to degrade 500 μg/L of phenol within a short time period. Strain DW-1 exhibited the highest biodegradation ability with low-concentration phenol as the sole carbon source. It is able to eliminate approximately 500 μg/L of phenol in less than 80 min, and the degradation rate reach about 98%. Strain DW-1 that immobilized on sterilized BAC also exhibited a good ability to remove phenol. Phenol removal could reach to approximately 20% with initial phenol concentration of 500 μg/L, and it could increase to approximately 86% with initial phenol concentration of 50 μg/L as shown in Supplementary Figure S3. According to the 16S rRNA gene sequencing, this strain is 99% similar to *Acinetobacter oleivorans* DR1. The phylogenetic tree indicates a strong association with the genus *Acinetobacter* as shown in Supplementary Figure S2. We can see that the genus *Acinetobacter* is also be detected in 50C by PCR-DGGE method corresponding to the band 4 (**Figure 2**).

### PCR Analysis of the Key Functional Genes of the Phenol Degradation Pathway

**FIGURE 6:**
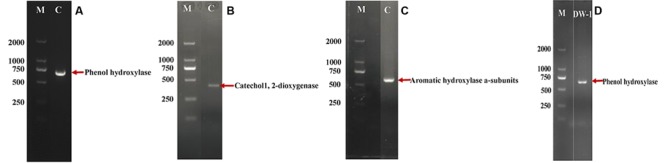
**PCR product analogs of the indicated catabolic genotypes obtained from DNA extracts of enrichment culture C on the 50th day during phenol acclimation and strain DW-1 using primers for Lph (A,D), 1,2-CTD (B) and TBMD (C).** The arrows denote amplicon fragment sizes.

To examine the metabolic pathway of the enriched culture, four key functional genes are detected using four pairs of primers (Lph, 1,2-CTD, 2,3-CTD, and TBMD), as describe in the section “Materials and Methods.” The four primer sets are for the amplification of genes that involve in the initial oxidation of phenol and the subsequent ring cleavage of catechol. After amplification, clearly defined bands were excised and sequenced. The sequences were compared using the BLAST software package, the results indicated that the enriched culture possessed two functional genes that belong to the catabolic pathway of phenol degradation. As shown in **Table 2**, the sequences were 97 and 83% similar to the Lph and 1,2-CTD genes, respectively. In addition, the bacterial consortium contained an aromatic hydroxylase gene, thus the bacterial consortium have potential to degrade aromatic compounds. The strain DW-1 had the most important phenol degradation gene: phenol hydroxylase. The results are shown in **Figure 6** and **Table 2**.

**Table 2:** Basic local alignment search tool (BLAST) results of the phenol degradation gene sequences in the final enrichment culture C and the strain DW-1.

Primer	Amplicon size(bp)	Nucleotide blast results	Similarity (%)	Accession number
Lph	575	*Acinetobacter oleivorans* DR1 phenol hydroxylase (CP002080)	97	KU512639
1,2-CTD	388	*Pseudomonas fluorescens* PF0-1 catechol 1,2- dioxygenase (CP000094)	83	KU512641
TBMD	640	*Azotobacter vinelandii* DJ Phenol hydroxylase subunit P3(CP001157)	91	KU512638
Lph	575	*Burkholderia* sp. 383 phenol hydroxylase (CP000152)	96	KU512640

## Discussion

### Acclimating the Phenol-degrading Community

The persistence of phenol in drinking water sources at toxic concentrations remains an important public health issue. The biodegradation of phenol by mixed culture has been investigated previously. Previous studies have reported on the phenol-degrading bacterial community in nutrient-rich environments such as lakes (Huang et al., 2014) and wastewater (Chakraborty et al., 2010). However, there has been no report about the impact of phenol on bacterial community structure in drinking water biofilters. During acclimation, phenol-resistant bacteria were favored. These bacteria may also have potential for phenol removal, which was studied and shown. In this study, the bacterial consortium degraded high concentrations of phenol over a short period of time under oligotrophic conditions, whereas a previous study found that a longer period of time was required to remove phenol at the same concentrations (Guieysse et al., 2001). Thus, the results of the present study suggest that bacteria from the BAC layer in drinking water have a strong potential for phenol removal.

### Characterization of the Bacterial Community Structure

Information on the phylogenetic composition of bacterial communities in drinking water biofilters remains scarce. In this study, the DGGE results that identified the main bacteria presenting in BAC from drinking water biofilters are shown in **Figure 2** and **Table 1**: *Campylobacter* sp., *Niastella* sp., *Deinococcus* sp., *Delftia* sp., *Achromobacter* sp., and *Agrobacterium* sp.. These taxa differed from those identified in previous studies as the dominant bacteria, namely, *Planctomyces, Flavobacterium, Microcystis, Fluviicola, Prosthecobacter*, and *Novosphingobium* (Liao et al., 2015b) as well as *Pseudomonas* sp., *B. subtilis*, *Nitrospira* sp., and an uncultured bacterium (Zhang et al., 2011) at the genus level. All of the main bacteria that identified in the present study have potential to degrade organic matters such as polyphenols (Reshma and Thanga, 2011), aniline, humic acid-absorbed phenanthrene and peptidoglycan (Liu et al., 2002; Vacca et al., 2005; Jørgensen et al., 2009), polychlorinated biphenyls (Ahmed and Focht, 1973) and BETX (Nielsen et al., 2006). Moreover, we found that within the same BAC-layer depth, the microbial community structures are highly similar according to the clustering analysis of the DGGE patterns. For example, the analysis suggested a high similarity (>80%) between communities from the same depth (**Figure 4** samples B and D). In addition, the microbial diversity of the ground-level BAC layer was markedly richer than that of the deeper BAC layer as shown in **Figure 2** (more bands in a lane correspond to a higher level of microbial diversity).

Recent work has investigated the bacteria that could reduce some organic matters, and also examined the change in microbial community due to the change in drinking water processes (Chung et al., 2008; Liao et al., 2012, 2013, 2014, 2015b; Wang et al., 2015). However, there is no information on the microbial communities in BAC filters that exposed to phenol, which could contribute to isolating the main indigenous bacteria that has excellent phenol-degrading ability in drinking water biofilters. Therefore, phenol biodegradation as a water treatment process could be enhanced by the exploration of bacterial community structure in BAC filters. A previous study have demonstrated that the species diversity of microbial communities in groundwater samples increased as the phenol levels decreased over a 9-month study period (Lin et al., 2007). However, in this study, after the acclimation period for 50 days, several new bacterial taxa appeared. For example, band 4 appeared after phenol acclimation, the result is consistent with the finding that γ-Proteobacteria was abundant during phenol treatment (Whiteley and Bailey, 2000). Numerous studies have also revealed various genus functions. For example, *Acidovorax* sp. was found to degrade phenol under low-oxygen conditions (Baek et al., 2003; Rizoulis et al., 2013). *Acinetobacter* sp. had never been isolated from oil-contaminated soil but can grow in diesel oil (Kang et al., 2011; Lee et al., 2012). *Sphingobium* sp. was detected in association with groundwater bioremediation and found capable of degrading polychlorophenol (Tiirola et al., 2002), bisphenol A (Toyama et al., 2012) and polycyclic aromatic hydrocarbons (Yuan et al., 2009). *Thiomonas* sp. isolated from sludge is a sulfur-oxidizing bacteria that is used in biological deodorisation (Chen et al., 2004). *Pseudomonas* sp., which also has been found in wastewater (Volkmann et al., 2007), is capable of degrading chloral hydrate by associate with other bacteria (Nomura et al., 2011).

Based on the phylogenetic affiliations that presented in **Table 1**, the band sequences are grouped into three phyla: *Proteobacteria*, *Bacteroidetes*, and *Deinococcus-Thermus*. A higher proportion of *Proteobacteria* (including α-, β-, γ-, and ε-*Proteobacteria*) was found in the BAC filter due to the increase in both α-*Proteobacteria* and β-*Proteobacteria*. These results complements those of previous studies that members of α-*Proteobacteria* dominate in BAC and are competitive under the low-nutrient concentrations (Liao et al., 2013; Lautenschlager et al., 2014). β-*Proteobacteria* has also been found to grow quickly and dominate in BAC filter eﬄuent (Liao et al., 2014). Similar results were obtained in this study, members of *Proteobacteria* are known to degrade complex organic compounds, such as chlorophenol, dichlorophenoxy acetic acid and polycyclic aromatic hydrocarbons (Rani et al., 2008). Members of *Bacteroidetes* were reported to contribute the main bacterial components in up-flow BAC filters in drinking water (Liao et al., 2012) and be capable of degrading polymeric organic matter. Finally, members of *Deinococcus-Thermus* are known to degrade alkane (Van Beilen and Funhoff, 2007). *Deinococcus-Thermus* was recently discovered as a main phylum present on BAC in drinking water. However, the bacterial community composition varies among BAC filters due to the differing nutrients, carbon substrates, and operational conditions (Boon et al., 2011). Thus, different drinking water pools yield different bacterial communities. To conclude, previous studies have shown that the bacterial community structure on BAC substrates change during phenol biodegradation. Our results indicate that the community structure considerably changed via acclimatization. **Figure 3** presents the results for the period preceding the acclimation process in rows 0A–0D, which fall into the same cluster but differ from the cluster in rows 50A–50D, approving the changes have occurred. **Figure 4** shows that there are no interaction effects between 0A–0D and 50A–50D, and these effects are in different areas. Therefore, these results reveal a significant difference between the before and after phenol acclimation.

### Isolation of Phenol-degrading Bacteria and Phenol Degradation Tests

The biodegradation of phenol by strains isolated from wastewater (Cheela et al., 2014; Nweke and Okpokwasili, 2014; Senthilvelan et al., 2014) and soil (Nweke and Okpokwasili, 2014) have been reported in previous studies, in which the bacterial strains were capable of degrading high concentrations of phenol (120–1,000 mg/L). However, phenol is usually present at much lower concentrations in drinking water source and finished water; the concentration of phenol in drinking water sources is about 100–5,000 μg/L (GB/T 5750 Standard examination methods for drinking water – General principles) due to the discharge of industrial and domestic wastewater. In some cases, the phenol concentration in drinking water sources could reach ppm level, since chemical leakage from ships or disorderly discharged sewage by manufacturers. In addition, the degradation rate and attenuation mechanism of phenol at these concentrations are unknown. So, it has practical significance to study the he biodegradation of phenol at low concentrations. Furthermore, it is also necessary to isolate and identify the indigenous microorganisms due to their harm-reducing properties of these organisms. In many cases, the isolated strains in drinking water biofilters have not been identified. The present study found that the strain DW-1 exhibited the most effective degradation of phenol at low concentration, which was 99% similar to *A. oleivorans* DR1, according to the phylogenetic analysis of 16S rRNA gene sequences. *Acinetobacter* sp. are known to degrade many pollutants, such as biphenyl, chlorinated biphenyl, atrazine (Singh et al., 2004), fenoxaprop-P-ethyl (Dong et al., 2015), polyethylene (Pramila and Ramesh, 2015), and pyrene (Yuan et al., 2014). And more importantly, *Acinetobacter* sp. has been found to be able to degrade phenol (Paller et al., 1995; Abu Hamed et al., 2004; Liu et al., 2009). In addition, according to the profiles of PCR-DGGE, the strain DW-1, which belongs to the genus *Acinetobacter*, matched well with the 16S rRNA gene sequence that retrieved from the DGGE band 4 (**Figure 2**). And the band 4 is the dominant bacteria after phenol acclimation. This indicates that the strain DW-1 that isolated from BAC filter could efficiently reduce phenol, and it could be one of the most important and potential phenol-degrading bacteria in this community. Moreover, the results of phenol degradation tests have proved above conclusion. Strain DW-1 that immobilized on GAC also exhibited a good ability to degrade phenol. From this perspective, the indigenous bacteria showed great reusable value for phenolic water remediation in DWTPs. Although preliminary results show that there are many methods have been used for remediating phenolic-bearing water, including reverse osmosis (Matsuura and Sourirajan, 1971; Sun et al., 1995), nanofiltration (López-Muñoz et al., 2009), ultraviolet radiation and advanced oxidation processes (Huber et al., 2003; Matilainen and Sillanpää, 2010), adsorption (Dąbrowski et al., 2005; Ali and Gupta, 2006), immobilized enzymes (Lante et al., 2000). However, these methods suffer from several drawbacks such as high capital, complexity, operating and maintenance costs, high energy input, difficulty in handling low-concentration phenol and toxic by products. Biological treatment, though slow under environmental conditions, is thought to be an important mechanism for the removal of low-concentration phenol, and it could directly degrade (Feist and Hegeman, 1969; Trojanowski et al., 1977), thus prevent the contamination from increasing in drinking water. The energy source for phenol decomposition was provided by itself. Moreover, reusing the indigenous bacteria to degrade phenol would not bring in alien species that may change the microbial ecology in drinking water treatment systems. And the indigenous bacteria always exhibit a good adaptability to the environment. Therefore, it is a cost-effective and highly efficient method to reuse native microorganisms for phenol removal in drinking water sources.

### PCR Analysis of the Key Functional Genes of the Phenol Degradation Pathway

The phenol biodegradation pathway reveal that the phenol-enriched culture produce phenol hydroxylase and catechol 1,2-dioxygenase, which indicate that the enrichment culture could degrade phenol by the orth-pathway. Meanwhile, many strains degrade phenol via meta-pathways, in which phenol hydroxylase and catechol 2, 3-dioxygenase are induced. In the ortho- and meta-pathways, catechol ring is decomposed into acetate, succinate, pyruvic acid, and acetaldehyde, as shown in Supplementary Figure S1. Strain DW-1 also produced phenol hydroxylase in the first step of phenol oxidation, which is considered to be the key metabolic reaction due to the aromatic ring-cleavage step is much easier after the first ring-cleavage oxidization. Therefore, strain DW-1 is important for phenol elimination in drinking water. This is the first report about the investigation for phenol biodegradation pathway, and the isolation of indigenous bacteria with the ability to degrade phenol in BAC filter in drinking water environment.

## Conclusion

This study is the first time to characterize the specific microbiome under trace phenol conditions in drinking water BAC filters by PCR-DGGE. The bacteria that achieved the highest phenol degradation efficiencies was isolated and characterized as *Acinetobacter* sp. The PCR-DGGE analysis documents the differences between before and after acclimation, and identifies the predominant bacteria in drinking water biofilters as *Delftia* sp., *Achromobacter* sp., and *Agrobacterium* sp.. In addition, indigenous bacteria possessed phenol hydroxylase, 1,2-dioxygenase and aromatic hydroxylase, which played important role in phenol biodegradation pathway, thus the indigenous bacterial diversity in drinking water biofilters has great potential in eliminating phenol. The strain DW-1with the fastest phenol-degrading rate was isolated and characterized as *Acinetobacter* sp., which can produce phenol hydroxylase, the most important oxidase, and it can completely degrade phenol (500 μg/L) within 80 min under oligotrophic conditions. And strain DW-1 that immobilized on sterilized BAC also exhibited a good ability to remove phenol. These results suggest that the strain DW-1 may have a great potential of being used as bioremediation in phenol-polluted drinking water. This study provides important baseline data for bacterial component that can degrade phenol, and offers an approach to investigate the special bacterial community and to recover functional microorganisms from drinking water biofilters in the future studies.

## Author Contributions

Conceived and designed the experiments: QW, JZ, and QG. Performed the experiments: QG and WG. Analyzed the data: QG and MS. Contributed reagents/materials/analysis tools: HW. Contributed to the writing of the manuscript: QG and QW.

## Conflict of Interest Statement

The authors declare that the research was conducted in the absence of any commercial or financial relationships that could be construed as a potential conflict of interest.

## Footnotes

1 http://www.ncbi.nlm.nih.gov
