# Supplementary material for: Community Analysis and Recovery of Phenol-degrading Bacteria from Drinking Water Biofilters
Source: Front Microbiol. 2016 Apr 12;7:495. doi: 10.3389/fmicb.2016.00495 (PMC4828441; doi:10.3389/fmicb.2016.00495)
Supplement: Supplementary file 1 [file Presentation_1.PDF]

Supplementary Material

Community analysis and characterization of phenol-degrading isolates in drinking water biofilters

Qihui Gu1, 2, Qingping Wu2*, Jumei Zhang2, Weipeng Guo2, Huiqing Wu2, Ming Sun2

*** Correspondence:** Qingping Wu: E-mail: wuqp203@163.com

Figure legends of supplementary materials

Fig.S1 Phenol metabolic pathways.

Fig.S2 A neighbour-joining tree that shows the phylogenetic relationships of strain DW-1 and closely related taxa. Scale changes of 0.5 distinguish each nucleotide position.

Fig.S3 Phenol removal of strain DW-1 immobilized on BAC under different initial phenol concentrations. Sterilized BAC in glass column as control. The mean values from triplicate experiments and the standard errors of the means, indicated by error bars, are shown.

Fig.S4 The samples were continuously acclimated with different concentrations of phenol in 50 days. (a), (b) and (c) are correspond to sample A, B and D, respectively. No phenol degradation occurred in autoclaved control bottles. The mean values for three active bottles and three control bottles were collected.
