# Supplementary figures and images for: Community Analysis and Recovery of Phenol-degrading Bacteria from Drinking Water Biofilters
Source: Front Microbiol. 2016 Apr 12;7:495. doi: 10.3389/fmicb.2016.00495 (PMC4828441; doi:10.3389/fmicb.2016.00495)

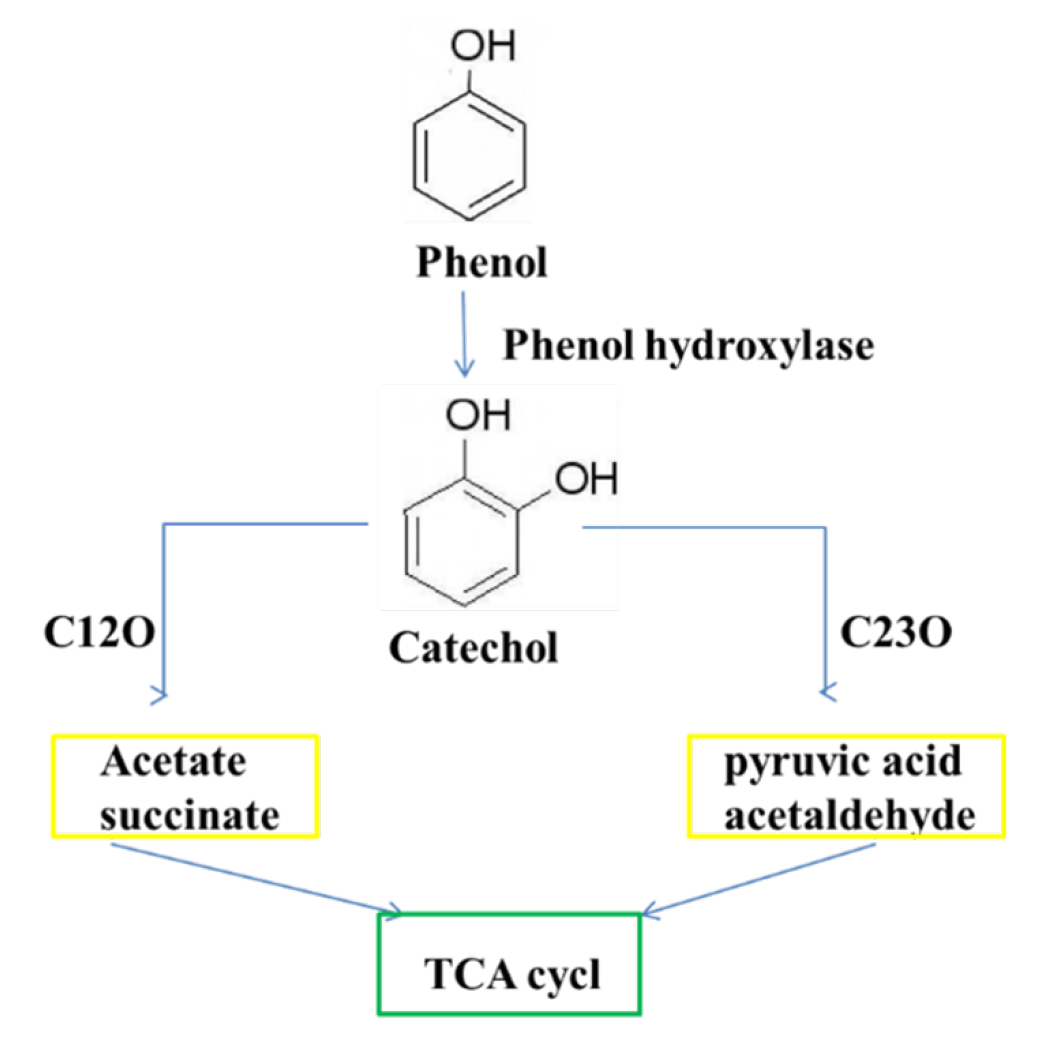

Supplement: Supplementary file 2 [file Image_1.TIF]

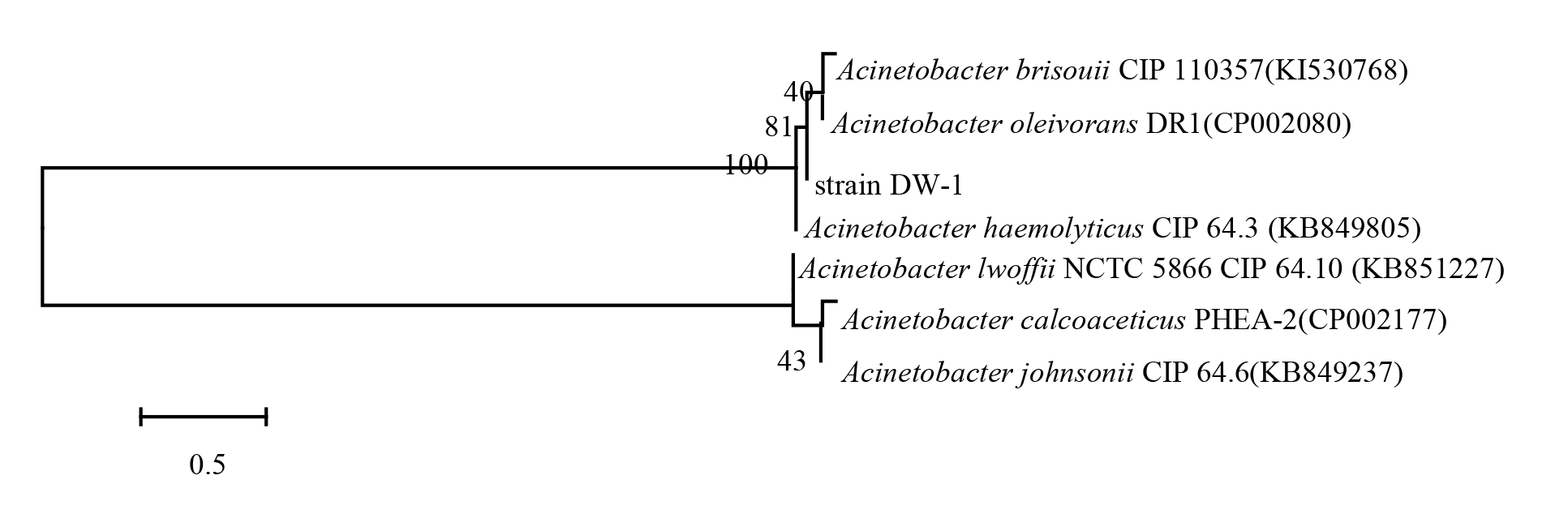

Supplement: Supplementary file 3 [file Image_2.TIF]

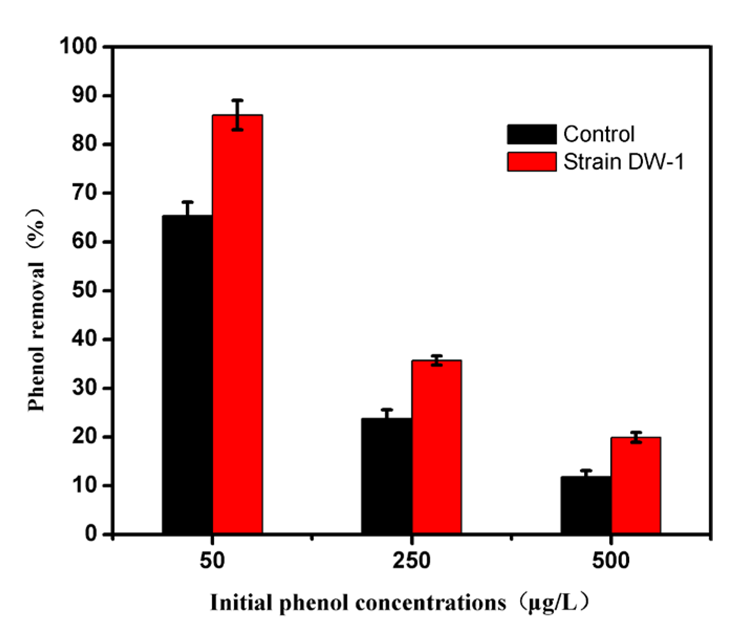

Supplement: Supplementary file 4 [file Image_3.TIF]

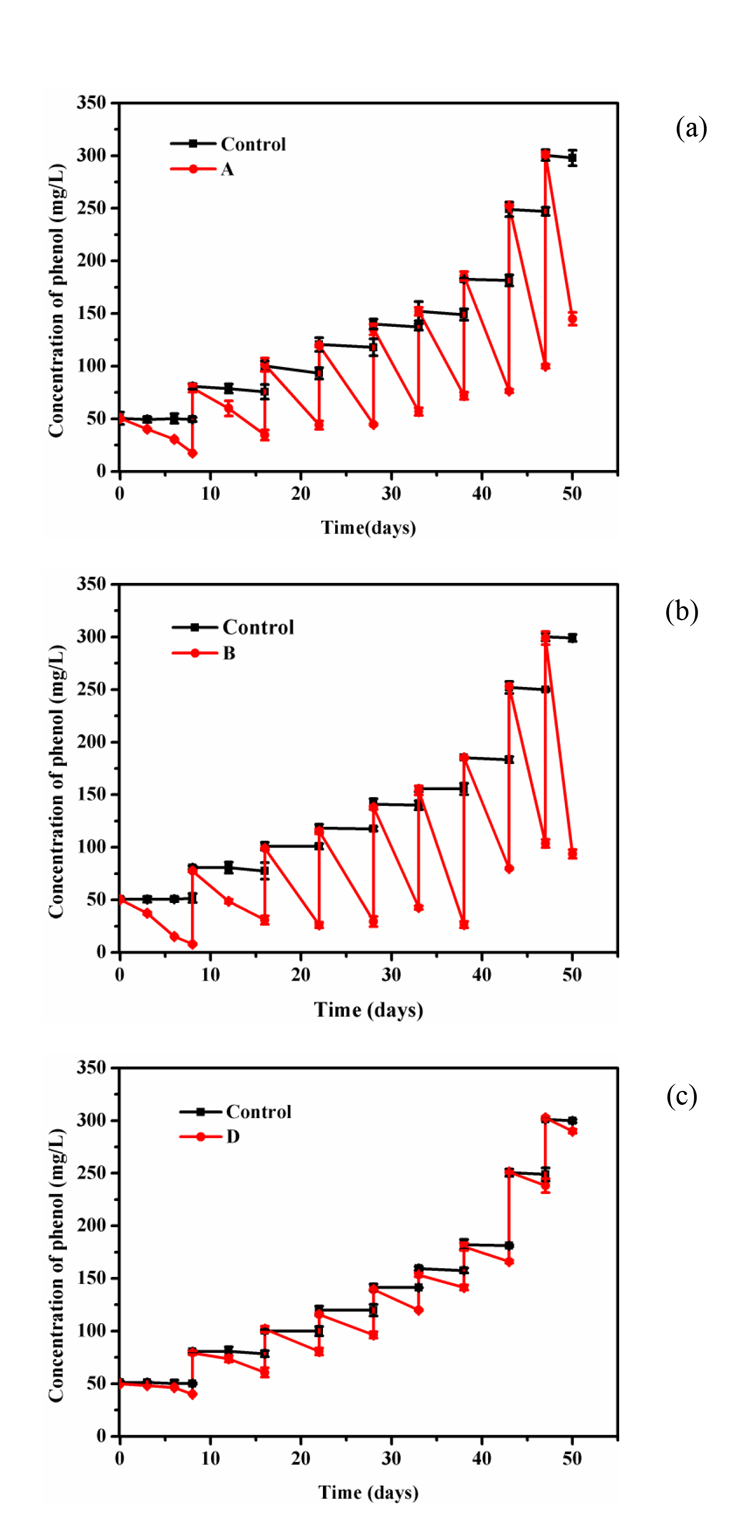

Supplement: Supplementary file 5 [file Image_4.TIF]
